# Going from microbial ecology to genome data and back: studies on a haloalkaliphilic bacterium isolated from Soap Lake, Washington State

**DOI:** 10.3389/fmicb.2014.00628

**Published:** 2014-11-19

**Authors:** Melanie R. Mormile

**Affiliations:** Department of Biological Sciences, Missouri University of Science and TechnologyRolla, MO, USA

**Keywords:** Soap Lake, *Halanaerobium hydrogeniformans*, alkaliphile, halotolerant, biohydrogen, genome analysis

## Abstract

Soap Lake is a meromictic, alkaline (∼pH 9.8) and saline (∼14–140 g liter^-1^) lake located in the semiarid area of eastern Washington State. Of note is the length of time it has been meromictic (at least 2000 years) and the extremely high sulfide level (∼140 mM) in its monimolimnion. As expected, the microbial ecology of this lake is greatly influenced by these conditions. A bacterium, *Halanaerobium hydrogeniformans*, was isolated from the mixolimnion region of this lake. *Halanaerobium hydrogeniformans* is a haloalkaliphilic bacterium capable of forming hydrogen from 5- and 6-carbon sugars derived from hemicellulose and cellulose. Due to its ability to produce hydrogen under saline and alkaline conditions, in amounts that rival genetically modified organisms, its genome was sequenced. This sequence data provides an opportunity to explore the unique metabolic capabilities of this organism, including the mechanisms for tolerating the extreme conditions of both high salinity and alkalinity of its environment.

## INTRODUCTION

Soap Lake is a meromictic, haloalkaline lake located in Washington State. It thought that the aerobic and anaerobic layers of this lake have not mixed in over 2000 years (Peyton and Yonge, 2002). The lake’s meromictic characteristic is due to the steep gradient in salt concentrations between the mixolimnion and the monimolimnion, 15 gL^-1^ and 140 gL^-1^, respectively (Sorokin et al., 2007), and the shape of the lake’s basin (Edmondson and Anderson, 1965). It is the terminal lake in the chain of lakes that formed in the Lower Grand Coulee during the Missoula Floods. This terminal lake has no surface inlets or outlets. The lack of outlets is the primary reason for the lake’s salinity (Anderson, 1958). Soap Lake’s water levels are supplied by water runoff from cliffs and plateaus surrounding the lake and from groundwater seepage, with evaporation as the main method for water loss (Anderson, 1958). The alkalinity of Soap Lake is maintained at a nearly constant pH of 9.8 in both the mixolimnion and the monimolimnion (Dimitriu et al., 2008). This alkalinity is controlled by the presence of carbonates and bicarbonates. The concentrations of carbonates in the mixolimnion of Soap Lake average around 8,500 mgL^-1^ and 24,000 mgL^-1^ in the monimolimnion. In comparison, the concentrations of bicarbonates in Soap Lake were always found to be lower than the carbonates with 2000 mgL^-1^ in the mixolimnion and 4,800 mgL^-1^ in the monimolimnion (Anderson, 1958).

This environment, due to its high salinity and alkalinity, impacts the microbial community in a number of ways. Though the pH of the environment is 9.8, it can be predicted that the internal pH values of the organisms present is lower. As such, alkaliphilic bacteria must be able to maintain homeostasis (Krulwich, 1995). In addition, there is a greater energy cost for the production of adenosine triphosphate (ATP) via chemiosmotic means under alkaline conditions (Krulwich et al., 2011). The organisms present also have to retain water in their cells and maintain osmotic homeostasis. They can achieve this by either using a “salting in” strategy or by using organic osmoregulatory compounds. The “salting in” process is typically used by Archaea while Bacteria tend to rely on osmoprotectant compounds.

A number of interesting and novel haloalkaliphilic bacteria have been isolated from Soap Lake. These bacteria include, *Ectothiorhodospira vacuolata* strain (Chadwick and Irgens, 1991), *Halomonas campisalis* (Mormile et al., 1999), *Nitrincola lacisaponensis* (Dimitriu et al., 2005), *Thiocapsa imhoffii* (Asao et al., 2007), *Bacillus* sp. strain SFB (Pollock et al., 2007), *Alkalitalea saponilacus* (Zhao and Chen, 2012), and ‘Candidatus *Heliomonas lunata’* strain SLH (Asao et al., 2012). *Halanaerobium hydrogeniformans* was isolated from an enrichment initially prepared for iron-reducing bacteria (Begemann et al., 2012). Though the bacterium is capable of iron-reduction (Paul et al., 2014), it can grow fermentatively on a variety of carbohydrates producing H_2_ in yields comparable to a *Clostridium paraputrificum* that was modified to overexpress a hydrogenase gene (Begemann et al., 2012). Due to *Halanaerobium hydrogeniformans*’ ability to produce notable amounts of H_2_ from sugars and its haloalkaliphilic characteristics, its genome was sequenced and annotated (Brown et al., 2011).

*Halanaerobium hydrogeniformans* is a Gram negative, non-motile, non-sporulating rod-shaped bacterium (Begemann et al., 2012). Its genome size is 2,613,116 bp and has a 33.1% G+C content (Brown et al., 2011). It also contains 2,391 candidate protein-encoding genes. In addition to biofuel applications, the availability of the genome sequence and annotation data of *Halanaerobium hydrogeniformans* enables the determination of the adaptations this organism possesses that facilitates it to thrive under the haloalkaline conditions found in Soap Lake.

## MATERIALS AND METHODS

*Halanaerobium hydrogeniformans’* genome data (Brown et al., 2011) was interrogated to gain information on the function of this bacterium’s genome. Information on candidate protein-encoding genes and RNA genes were obtained by using the integrated microbial genomes (IMG) system (Markowitz et al., 2012). BioCyc databases and pathway tools were also used (Caspi et al., 2010). Another sequenced *Halanaerobium*, *Halanaerobium praevalens* GSL^T^ (Ivanova et al., 2011) a non-alkaliphilic bacterium, was used as a comparator organism. *Halanaerobium praevalens* GSL^T^ was first isolated from the sediments of the Great Salt Lake in Utah (Zeikus et al., 1983). Similar amino acid sequences were determined by performing protein BLAST searches (Altschul et al., 1997). The complete genome of *Halanaerobium hydrogeniformans* has been deposed in NCBI Genomes with accession number NC_014654.

## RESULTS AND DISCUSSION

### GENOME PROPERTIES

**FIGURE 1:**
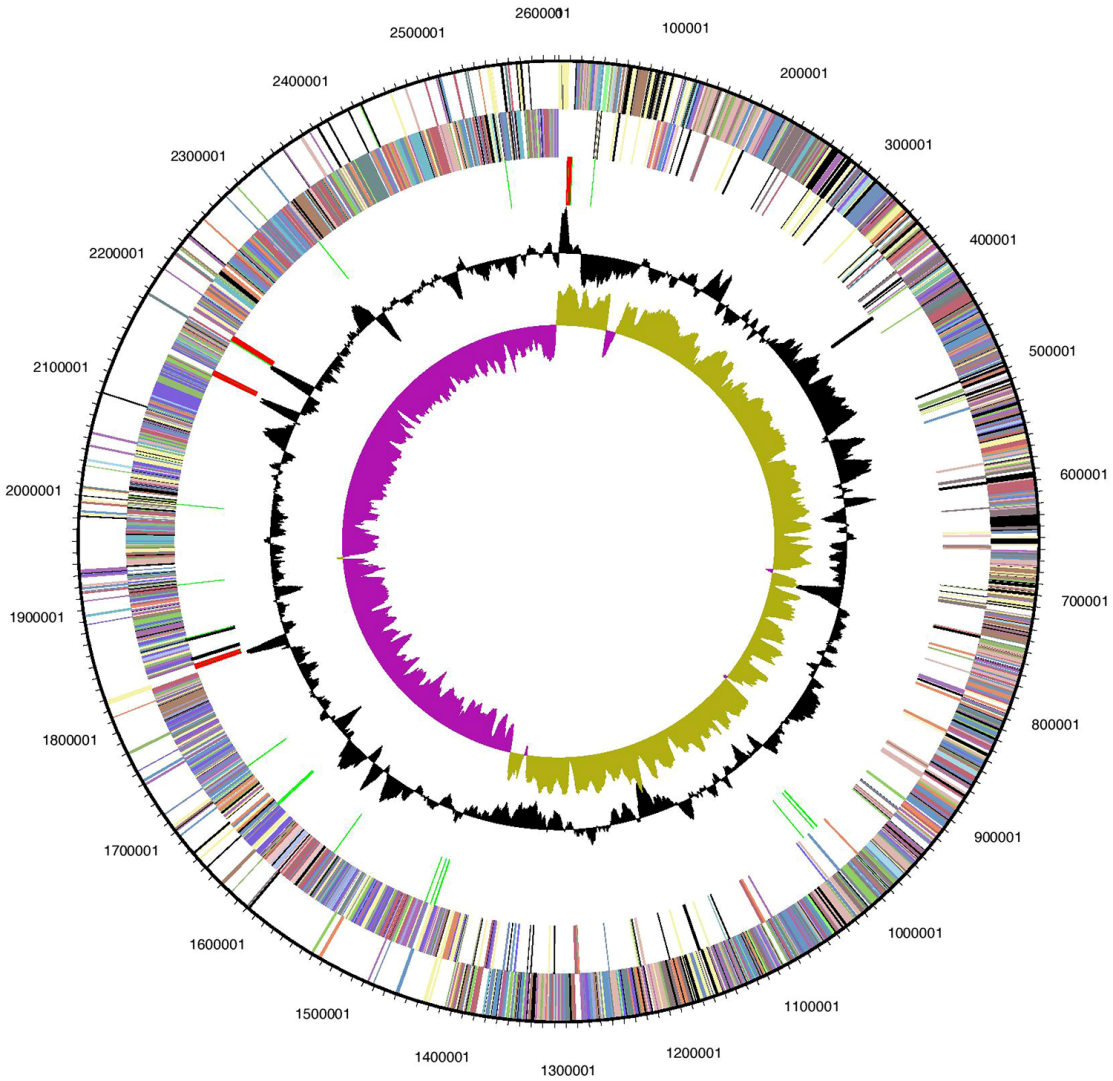
**Graphical circular map of *Halanaerobium hydrogeniformans*’ genome.** From the outside to the center: genes of the forward strand (color by COG categories), genes on reverse strand (color by COG categories), RNA genes (tRNAs green, rRNAs red, other RNAs black), GC content, GC skew. Image generated from IMG (Markowitz et al., 2012).

Of the 2391 candidate protein-encoding genes, there are 1867 with function predictions in the genome (**Table 1**). Four 5S rRNA, 16S rRNA, and 23S rRNA genes each are present as are 57 tRNA genes. There are 2082 genes assigned to clusters of orthologous groups (COGs). Interestingly, approximately 25% of the protein-encoding genes are for transmembrane proteins. The distribution of the genes into COG functional categories is provided in **Figure 1** and **Table 2**. The gene count for the different Kyoto Encyclopedia of Genes and Genomes (KEGG) categories is similar between *Halanaerobium hydrogeniformans* and *Halanaerobium praevalens* GSL^T^ except for a few categories (**Table 2**). *Halanaerobium praevalens* GSL^T^ only has a gene count of 85 for amino acid metabolism while *Halanaerobium hydrogeniformans* has 138. *Halanaerobium praevalens* GSL^T^ also has lower gene counts for the KEGG categories of metabolism and metabolism of cofactors and vitamins. On the other hand, *Halanaerobium hydrogeniformans* has a much lower gene count for KEGG category cell motility. Though both of these organisms are not considered to be motile, there are strains of *Halanaerobium praevalens* GSL^T^ that are (Kobayashi et al., 2000 and Eder et al., 2001).

**Table 1:** Genome statistics.

	Number
Total number of bases	2,613,117
Number of DNA coding bases	2,286,541
G+C percentage	33.16
Contigs	1
Total number of genes	2463
Number of protein coding genes	2391
Pseudo genes	96
5S rRNA genes	4
16S rRNA genes	4
23S rRNA genes	4
tRNA genes	57
Genes with function prediction	1867
Genes assigned to clusters of orthologous groups (COGs)	2082
Genes coding transmembrane proteins	624

**Table 2:** Number of genes associated with the general COG functional categories.

KEGG Category	*Halanaerobium hydrogeniformans* gene count	*Halanaerobium praevalens* gene count
Amino acid metabolism	138	85
Biosynthesis of other secondary metabolites	15	8
Carbohydrate metabolism	171	137
Cell motility	5	59
Energy metabolism	93	82
Folding, sorting, and degradation	32	30
Glycan biosynthesis and metabolism	25	28
Lipid metabolism	51	39
Membrane transport	95	83
Metabolism	486	375
Metabolism of cofactors and vitamins	110	83
Metabolism of other amino acids	24	32
Metabolism of terpenoids and polyketides	20	17
Nucleotide metabolism	77	81
Replication and repair	41	39
Signal transduction	39	53
Transcription	4	4
Translation	77	80
Transport and catabolism	2	1
Xenobiotics biodegradation and metabolism	25	27

### METABOLIC CAPABILITIES

**FIGURE 2:**
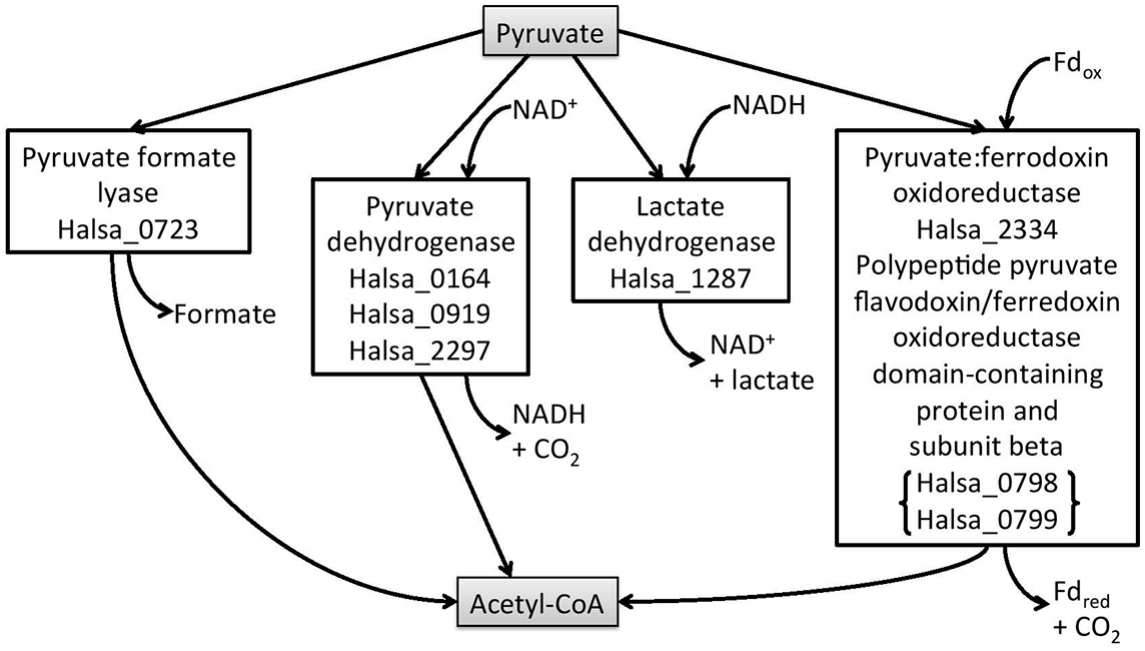
***Halanaerobium hydrogeniformans*’ possible metabolic routes from pyruvate.** The putative genes are given for each of the enzymes in the boxes.

*Halanaerobium hydrogeniformans* has 20% of its genes in the COG category of metabolism and 7% of its genes in the carbohydrate category. Thus, it is not surprising that *Halanaerobium hydrogeniformans* is capable of growth on a number of sugars derived from cellulose and hemicellulose (Begemann et al., 2012). When grown on cellobiose, biomass is produced along with fermentation products, such as formate, acetate, and hydrogen (Begemann et al., 2012). By considering the annotated genome, it should be possible to determine the putative pathway from cellobiose to hydrogen. Cellobiose can be brought into the cell by a putative phosphotransferase system (PTS) lactose/cellobiose-specific transporter subunit IIB (gene designated as Halsa_0653). Once inside the cell, cellobiose would be cleaved and enter the Embden-Meyerhof pathway of glycolysis with the formation of pyruvate. The enzymes for this pathway are present in *Halanaerobium Hydrogeniformans*^1^. Once formed, there are a number of possible fates for pyruvate. A putative pyruvate formate-lyase catalyzes pyruvate and coenzyme A to form formate and acetyl Co A (Halsa_0723). A possible fate for formate is to be broken down into CO_2_ and H_2_ by formate-hydrogen lyase (**Figure 2**). However, there was no gene identified that would code for the enzyme, formate-hydrogen lyase. As reported earlier, *Halanaerobium hydrogeniformans* does accumulate formate (Begemann et al., 2012). Thus, it is unlikely that this organism is forming hydrogen from formate. *Halanaerobium praevalens* GSL^T^ does not appear to possess this enzyme either. However, formate that is released by these fermentative organisms can be used by sulfate-reducing prokaryotes present in Soap Lake (Dimitriu et al., 2008).

*Halanaerobium hydrogeniformans*’ genome possesses an *ldh* gene, indicating that lactate dehydrogenase should also be present (Halsa_1287). However, lactate has not been detected as a metabolic product from this organism. It is interesting to note that many fermentative organisms possess *ldh* genes (Carere et al., 2012). However, only a few, such as *Bacillus cereus*, had been found to produce lactate in high yields.

**FIGURE 3:**
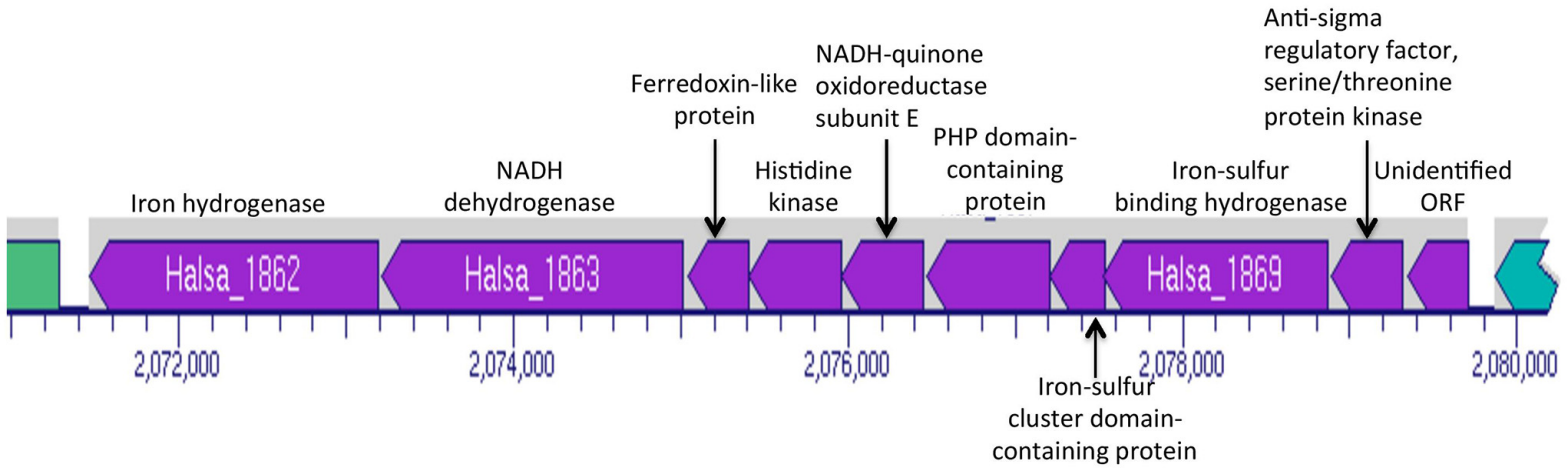
**Portion of *Halanaerobium hydrogeniformans’* genome map indicating the genes in the same operon as the putative hydrogenase, Halsa_1862.** Image generated from BioCyc (Caspi et al., 2010).

*Halanaerobium hydrogeniformans* appears to possess three putative pyruvate dehydrogenase genes (Halsa_0164, Halsa_0919, and Halsa_2297; **Figure 2**). Other genera, *Caldicellulosiruptor*, *Clostridia*, and *Thermoanaerobacter*, also possess putative *pdh* genes but there has been no evidence for functional enzyme production (Carere et al., 2012). *Halanaerobium hydrogeniformans* possesses a gene for the formation of pyruvate:ferredoxin oxidoreductase (Halsa_2334) as well as two genes that encode a polypeptide pyruvate flavodoxin/ferredoxin oxidoreductase domain-containing protein and subunit beta (Halsa_0798 and Halsa_0799). Furthermore, it possesses two genes, Halsa_1768 and Halsa_1862 that encode for iron hydrogenases. Halsa_1862 is part of a putative operon that includes a NADH dehydrogenase (Halsa_1863), a ferredoxin-like protein (Halsa_1864), a histidine kinase (Halsa_1865), NADH-quinone oxidoreductase subunit E (Halsa_1866), PHP domain-containing protein (Halsa_1867), an iron-sulfur binding hydrogenase (Halsa_1868), an iron-sulfur cluster domain-containing protein (Halsa_1869), an anti-sigma regulatory factor, serine/threonine protein kinase (Halsa_1870), and an unidentified open reading frames (ORF; Halsa_1871; **Figure 3**). The organism’s ability to produce substantial amounts of H_2_, 2.3 hydrogen molar yield from cellobiose, (Begemann et al., 2012) is of interest as a possible biofuel-producing organism.

It is likely that fermenters such as *Halanaerobium hydrogeniformans*, has a role in interspecies hydrogen transfer in the Soap Lake ecosystem. For example, sulfate- and iron-reducing bacteria were found in the sediments of Soap Lake (Dimitriu et al., 2008) and these organisms can serve as sinks for the H_2_ produced (Jones et al., 1998). However, there have been limited studies on interspecies hydrogen transfer in hypersaline environments. In our own studies, when H_2_ and CO_2_ were provided as substrates, low numbers of methanogens were detected in the sediments and monimolimnion of Soap Lake while no methanogens were detected in the mixolimnion and chemocline (Dimitriu et al., 2008). Due to thermodynamic constraints (-34 kJ/mol H_2_; Oren, 1999), autotrophic methanogenesis is unlikely to occur, especially in environments with large amounts of sulfate present, such as Soap Lake. Sulfate reduction with H_2_ is slightly more thermodynamically favorable than methanogenesis in hypersaline environments (Oren, 2010). In fact, hydrogenotrophic sulfate reducers have been reported from the hypersaline soda lakes of the Kulunda Steppe in southeastern Siberia in Russia (Foti et al., 2007). The first report of interspecies hydrogen transfer possible in hypersaline soda lakes involved a hydrogenotrophic sulfate-reducing bacterium, *Desulfohalobium retbaense*, was found to utilize the H_2_ produced by two species of *Halanaerobium*, *Halanaerobium saccharolytica* subsp. *Senegalense,* and *Halanaerobium* sp. strain FR1H from glycerol fermentation (Cayol et al., 2002). When *Desulfohalobium retbaense* was present as an H_2_-scavenger, glycerol consumption increased and H_2_ concentrations approached or were at undetectable amounts.

From early on, it was recognized that glycerol was a major carbon source in saline lakes (Borowitzka, 1981). Glycerol is produced as an osmoregulatory solute by organisms such as green alga, *Dunaliella salina* (Oren, 1993). Not only can glycerol be released from lysed cells but can also leak from healthy cells (Bardavid et al., 2008). This source of carbon can be used by halophilic aerobic prokaryotes, such as *Haloquadratum* and *Salinibacter*. These aerobic bacteria oxidize glycerol incompletely with excretion of products such as acetic acid, lactic acid, and pyruvic acid (Oren, 2008). Other microorganisms present in these hypersaline environments can subsequently use these products. When a cell takes up glycerol, the glycerol can be converted into dihydroxyacetone and then integrated into pyruvate metabolism, resulting in the products listed above. Glycerol can also be converted into 1,3-propanediol to replenish NAD^+^ from NADH_2_ resulting when glycerol is oxidized to dihydroxyacetone and dihydroxyacetone phosphate is oxidized to phosphoenolpyruvate. Much of the NADH_2_ produced is recycled to NAD^+^ through the formation of fermentation end products, such as ethanol, acetate, and butyrate. However, some NAD^+^ must be replenished through an alternate pathway (Zeng, 1996). Excess glycerol can be shunted into the 1,3-propanediol production pathway where NADH_2_ is re-oxidized to form 1,3-propanediol. This metabolism is present in *Halanaerobium hydrogeniformans* (Roush et al., 2014).

The metabolism of glycerol is of interest not only for its ecological role as a source of carbon in saline lakes but also for the formation of commodity compounds, such as 1,3-propanediol. Glycerol is formed as a byproduct during biodiesel production (Thompson and He, 2006). The first step in the conversion of glycerol to 1,3-propanediol is the removal of a water molecule from glycerol by the enzyme glycerol dehydratase. This step creates the intermediate 3-hydroxypropanal. Next, the enzyme 1,3-propanediol dehydrogenase, oxidizes NADH_2_ to form 1,3-propanediol, replenishing the NAD^+^ needed by the cell for normal metabolism (Zeng, 1996). The genome of *Halanaerobium hydrogeniforman*s revealed that it possessed the possibility of this metabolism^2^. The genes that it possesses that can possibly contribute to this pathway are Halsa_0984 (a putative glycerol dehydratase), Halsa_0672 (a putative 1,3-propanediol dehydrogenase), and Halsa_2285 (another putative 1,3-propanediol dehydrogenase). It was determined experimentally that *Halanaerobium hydrogeniformans* is capable of forming 1,3-propanediol from glycerol. After a 5-day incubation with 30 mM glycerol and pH 11 and 7% NaCl conditions, *Halanaerobium hydrogeniformans* was able to convert 31.5% of the glycerol to 1,3-propanediol. When B_12_ was provided at concentrations from 25 to 100 μg/L, glycerol to 1,3-propanediol conversion ranged from 59.1 to 60.3% (Roush, 2013).

Glycine betaine is another osmoregulatory compound found in hypersaline environments (Welsh, 2000). *Halanaerobium hydrogeniformans* possesses an ATP-binding cassette (ABC) transporter, Halsa_1783, that can possibly bring this compound into the cell. Not only can this compound be used as an osmoregulatory compound but can be a potential source of energy and carbon for the cell. Glycine betaine could possibly be used in the Stickland reaction with the amino acid, serine, as observed in *Halanaerobacter salinarius* (Mounté et al., 1999).

### MOBILE DNA

*Halanaerobium hydrogeniformans*’ genome was interrogated by using IMG to determine the most abundant COGs genes present. The most abundant COG genes in this genome were found to be transposases (**Table 3**). This should not come as a surprise as Aziz et al. (2010) found that transposases are both ubiquitous and abundant in both genomes and metagenome libraries. They determined the average number of transposases possessed across known genomes to be 38 per genome. *Halanaerobium hydrogeniformans* contains 72 annotated transposase genes (**Table 3**). In comparison, *Halanaerobium praevalens* GSL^T^ was found to possess 20 annotated transposase genes. Tranposase enzymes are responsible for the excision and movement of DNA segments within a chromosome. Transposase-encoding genes are flanked with insertion sequences (IS). These IS are short, inverted terminal repeats. Previously, it was thought that IS segments of DNA were selfish or parasitic (Orgel and Crick, 1980). However, it is now thought that transposable elements convey selective advantages to their hosts. These advantages can include the mobilization and/or activation of beneficial genes (Nowacki et al., 2009) or to generate phenotypic diversity (Brazelton and Baross, 2009). However, there are costs, such as transposon-induced mutations, that need to be balanced by the organisms (Aziz et al., 2010).

**Table 3:** Most abundant COG genes identified in *Halanaerobium hydrogeniformans’* genome.

COG ID	COG Name	*Halanaerobium hydrogeniformans*	*Halanaerobium praevalens* GSL^T^
2801	Transposase and inactivated derivatives	22	6
2963	Transposase and inactivated derivatives	20	6
2206	HD-GYP domain	14	9
2826	Transposase and inactivated derivatives, IS30 family	12	2
0438	Glycosyltransferase	11	9
3328	Transposase and inactivated derivatives	10	0
1028	Dehydrogenases with different specificities (related to short-chain alcohol dehydrogenases)	9	2
3437	Response regulator containing a CheY-like receiver domain and an HD-GYP domain	9	3
0675	Transposase and inactivated derivatives	8	6
0747	ABC-type dipeptide transport system, periplasmic component	7	6
1309	Transcriptional regulator	8	7
2199	FOG: GGDEF domain	8	9

*Halanaerobium praevalens GSL^T^ number of genes for each COG ID is also provided.*

A further breakdown of the transposases in *Halanaerobium hydrogeniformans* reveals that eight IS families are present in this genome (**Table 4**). IS families are based upon similarities and differences in structure, organization, and the nucleotide and protein sequence relationships (Mahillon and Chandler, 1998). For example, the IS*3* family is characterized by having lengths between 1,200 and 1,550 base pairs (bp) and inverted terminal repeats of 20 to 40 bp (Mahillon and Chandler, 1998). Interestingly, these sequences generally have two consecutive and partially overlapping ORF, *orfA* and *orfB*. These mobile segments of DNA transposes through a circular intermediate. Of the IS families identified in *Halanaerobium hydrogeniformans*’ genome, the only other IS family present that possesses more than one orf is IS*21*. The IS*21* family has two orfs, a long upstream frame, *istA*, and a shorter downstream frame, *istB*. These two proteins carry several blocks of highly conserved residues (Mahillon and Chandler, 1998). Work is currently being done by Ron Frank, Missouri S&T, to determine if the putative transposases are active in *Halanaerobium hydrogeniformans*. If so, it is suspected that these genes are can become mobile and potentially activate beneficial genes to increase the fitness of this organism to tolerate environmental pressures (Aziz et al., 2010) that are present in Soap Lake.

**Table 4:** Number of annotated genes in insertion sequence families present in *Halanaerobium hydrogeniformans*.

Insertion sequence family	Number of genes present
IS*3*	22
IS*30*	11
IS*256*	8
IS*200*/IS*605*	7
IS*4*	3
IS*6*	3
IS*21*	2
IS*91*	1

### CYCLIC-di-GMP

The second most numerous group of identified genes in the *Halanaerobium hydrogeniformans*’ genome are the HD-GYP domain genes of COGs 2206 and 3437 (**Table 3**). In addition, there are eight genes identified as belonging in COG 2199 of the FOG: GGDEF domain. The GGDEF domain encodes for enzymes that produce cyclic-di-GMP, a ubiquitous second messenger in bacteria (Jenal and Malone, 2006). It is involved in cell signaling, exopolysaccharide formation, attachment, and biofilm production. The HD-GYP domain genes encode for diguanylate cyclase and metal dependent phosphohydrolase, an enzyme responsible for producing cyclic-di-GMP and it requires the presence of divalent cations, most likely Mg^2+^ or Mn^2+^ (Castiglione et al., 2011). Previous analysis performed indicates that both of these metals, Mg^2+^ and Mn^2+^, 8,170.0 and 404.0 mg/kg dry weight, respectively, are present in the sediment of Soap Lake (Sigrid Penrod, personal communication). In comparing *Halanaerobium hydrogeniformans’* genome with *Halanaerobium praevalens* GSL^T^’s, only *Halanaerobium hydrogeniformans*’ genome possesses genes for diguanylate cyclase with metal dependent phosphohydrolase. Thus far, only a few environmental signals have been identified that regulate cyclic di-GMP-mediated signaling pathways (Römling et al., 2013), and none are know for *Halanaerobium*. *Halanaerobium hydrogeniformans* forms mucous-like mats in cultures that are not vigorously shaken (Begemann et al., 2012). One possible role this set of putative genes may play is the formation of these mats.

### GLYCOSYLTRANSFERASES

There is evidence for the occurrence of glycosyltransferases, COG 0438 (**Table 3**). Nine of the 11 putative genes in *Halanaerobium hydrogeniformans* encode for glycosyl transferase group 1 enzymes. There is one putative sucrose-phosphate synthase (Halsa_0772) and one hypothetical protein (Halsa_0632). These enzymes are defined by the utilization of an activated donor sugar group substrate that contains a phosphate leaving group (Lairson et al., 2008). They are involved in the biosynthesis of cell walls, membranes, and envelop biogenesis. Specifically, these enzymes catalyze the first step in the sucrose synthesis pathway and are thought to play a role in osmotic stress protection (Chua et al., 2008). *Halanaerobium hydrogeniformans*’ Halsa_0772 gene has a 74% identity to a sucrose-phosphate synthase that is present in *Halanaerobium praevalens* GSL^T,^ indicating a common mechanism for osmotic stress protection.

### SHORT-CHAIN DEHYDROGENASES/REDUCTASES (SDRs)

Nine putative genes in COG 1028 were found in *Halanaerobium hydrogeniformans*’ genome. Only two were found in *Halanaerobium praevalens* GSL^T^’s genome. These genes encode for short-chain dehydrogenases/reductases (SDRs) with different specificities. This super family of enzymes catalyze a variety of NAD(P)(H) oxidation/reduction reactions (Kallberg et al., 2002). These enzymes are also recognized to catalyze the metabolism of steroids, cofactors, carbohydrates, lipids, aromatic compounds, and amino acids, and act in redox sensing. They are also associated with biotin metabolism and fatty acid biosynthesis and metabolism. There hasn’t been much research performed on this family of enzymes in extremophilic bacteria. The research that has been focused on characterizing these enzymes from extremophilic organisms has been on thermophilic prokaryotes such as, *Thermus thermophiles* HB8 (Asada et al., 2009), *Sulfolobus acidocaldarius* (Pennacchio et al., 2010), and *Thermococcus sibiricus* (Stekhanova et al., 2010).

### ABC TRANSPORTERS

There are a number of ATP-binding cassettes (ABC) transporters represented in *Halanaerobium hydrogeniformans*’ genome. Of these, seven COG 0747 putative genes have been identified, Halsa_0302, Halsa_0968, Halsa_1628, Halsa_1745, Halsa_2053, Halsa_2146, and Halsa_2227 (**Table 3**). These genes encode for ABC-type nickel/dipeptide/oligopeptide periplasmic transport systems (Tam and Saier, 1993). Nickel is required for five types of enzymes; urease, hydrogenase, carbon monoxide dehydrogenase, methyl-*S*-coenzyme M reductase, and one class of superoxide dismutase (Hausinger, 1997). *Halanaerobium hydrogeniformans* does not appear to possess any of these enzymes. However, there are 524 genes that have been identified as hypothetical proteins and have no assigned functions. Thus far, only two possible hydrogenases, Halsa_1768 and Halsa_1862, have been identified. These are both Fe-only hydrogenases. It will be interesting to determine the concentration of nickel that is required by the organism as well as to determine if there are nickel-requiring enzymes present.

The protein-coding genes that were connected to membrane transport KEGG pathways were explored through IMG. These genes can indicate what is needed and utilized by the bacterium. For example, there are numerous genes that encode for iron uptake proteins. Iron III can possibly be taken up by proteins encoded by *AfuA* (Halsa_2074), *AfuB* (Halsa_2073), and *AfuC* (Halsa_2072). Siderophore-mediated transport of iron complexes are likely in this bacterium. These proteins can possibly be encoded by *FhuD* (Halsa_2140, Halsa_2186, Halsa_2212, and Halsa_2233), *FhuB* (Halsa_1986, Halsa_2185, Halsa_2211, and Halsa_2232), and *FhuC* (Halsa_1985, Halsa_2184, Halsa_2210, and Halsa_2231). FhuD is a periplasmic protein and FhuB and FhuC are cytoplasmic membrane-associated proteins responsible for siderophore-mediated iron transport (Katoh et al., 2001). It appears that *Halanaerobium hydrogeniformans* also possesses genes for proteins responsible to taking up another metal, tungstate. *TupA* (Halsa_2175), *TupB* (Halsa_2174), and *TupC* (Halsa_2173) were each found to be present. These genes do not appear to be present in *Halanaerobium praevalens* GSL^T^. Zinc is another metal that is possibly taken up by *Halanaerobium hydrogeniformans*. *ZnuA* (Halsa_0273), *ZnuB* (Halsa_0275), and *ZnuC* (Halsa_0274) were found in the bacterium’s genome.

*Halanaerobium hydrogeniformans*’ ability to utilize various carbon sources can be inferred by the transporters that it contains. Halsa_1981 was identified as possibly being involved with uptake of glucose/mannose (*MalK*), maltose/maltodextrin (*MalK*), galactose oligomer/maltooligosaccharide (*MsmX*), arabinooligosaccharide (*MsmX*), raffinose/stachyose/melibiose (*MsmK*), sorbitol/mannitol (*SmoK*), α-glucoside (*AglK*), cellobiose (*MsiK*), and chitobiose (*MsiK*). In addition to Halsa_1981, other genes are present that could encode for other carbon-intake ABC transporters. A total of 10 putative genes for ABC transporters for ribose/autoinducer 2/D-xylose, *RbsB*, *RbsC*, and *RbsA*, were identified. The genes, UgpB, UgpA, and UgpE, responsible for sn-glycerol 3-phosphate uptake were also found. Currently, the range of sources of carbon is unknown for *Halanaerobium hydrogeniformans*. Previous studies have demonstrated that the bacterium can use glucose, cellobiose, ribose, xylose, arabinose, galatose, and mannose (Begemann et al., 2012).

Glycerol can be used as either a carbon source or as an osmoprotectant (Oren, 1993). *Halanaerobium hydrogeniformans* possesses the genes, *OpuBB* and *OpuBA*, that are putative osmoprotectant ABC transport genes. In addition, it has putative trehalose/maltose ABC transport genes, *ThuE*, *ThuF*, and *ThuG*. Trehalose is considered a universal stress molecule and can serve as an osmoprotectant and in *Chromohalobacter salexigens*, it can serve to protect against temperature extremes (Reina-Bueno et al., 2012). However, trehalose was not confirmed to protect against desiccation. *Halanaerobium hydrogeniformans* does appear to have a mechanism to protect itself against desiccation. When grown with little or no agitation, it grows in an opaque mass (Begemann et al., 2012). It possesses an operon that contains a capsular exopolysaccharide family protein (Halsa_0553), a lipopolysaccharide biosynthesis protein (Halsa_0554), a polysaccharide export protein (Halsa_0555), and a PHP domain-containing protein (Halsa_0556). Thus, *Halanaerobium hydrogeniformans* appears to be capable of protecting itself against osmotic and desiccation pressures.

### PHOSPHOTRANSFERASE SYSTEMS (PTSs)

In addition to the ABC transport systems, *Halanaerobium hydrogeniformans* has numerous PTSs to bring in sources of carbon. Glucose, maltose, arbutin/salicin, *N*-acetyl muramic acid, and trehalose can be brought into a cell with the Crr kinase protein (Halsa_0150 and Halsa_1861). *N*-acetyl-D-glucosamine can possibly be brought into the cell with NagE (Halsa_0149). Proteins encoded by *CelA* (Halsa_0141), *CelB* (Halsa_0142), and *CelC* (Halsa_0143), could bring cellobiose into the cell. Putative genes for mannitol (*MtlA*), sorbitol (*SrlA*, *SrlE*, *SrlB*), galactitol (*GatA*, *GatB*, *GatC*), and fructose (*FruA*) are also present.

*Halanaerobium hydrogeniformans* has two putative nitrogen-related PTS genes, Halsa_0019 and Halsa_2283. Nitrogen-related PTS genes are found in Gram-negative bacteria, can regulate carbon and nitrogen metabolism, are required for virulence by some bacteria, and can play a role in potassium homeostasis (Pflüger-Grau and Görke, 2010). Halsa_0019 is likely to be involved with the regulation of fructose metabolism. Halsa_0020 is a putative gene for *FruA*, a fructose PTS, and Halsa_0018 is a putative 1-phosphofructokinase. In addition, when a BLAST search was performed on the amino acid sequence encoded by Halsa_0019, a 79 and 76% identity was found with a fructose-specific PTS from *Halanaerobium saccharolyticum* and *Halanaerobium praevalens*, respectively. The role for Halsa_2283 isn’t as apparent as for Halsa_0019. The gene in the same operon, Halsa_2284, was not identified. In addition, when a BLAST search was performed on the amino acid sequence encoded by Halsa_2283, only a 54% identity was found for a fructose-specific PTS from *Halanaerobium saccharolyticum*.

### OTHER TRANSPORT SYSTEMS

Being bacterial and not archaeal, one of the intriguing aspects of the Halanaerobiales order is that they use a “salting in” mechanism to protect themselves against osmotic shock (Detkova and Boltyanskaya, 2007). *Halanaerobium hydrogeniformans* possesses putative genes that possibly encode for TrkA-C domain containing proteins (Halsa_0281, Halsa_0709, and Halsa_1061) and TrkA-N domain containing proteins (Halsa_0737, Halsa_1057, Halsa_1056, Halsa_1352, and Halsa_1257). These genes are responsible for potassium ion transport into the cell. In addition, there are a number of putative symporters for the cell. These include a putative sodium/dicarboxylate symporter (Halsa_0959), sodium/sulfate symporter (Halsa_1097), and sodium/proline symporter (Halsa_1726). It is interesting to note that these symporters would bring sodium into the cell. There are also putative Na^+^/H^+^ antiporters present. These antiporters would remove sodium from the cell while bringing in protons and contributing to the pH homeostasis of the cell (Janto et al., 2011). Halsa_0468, Halsa_1158, Halsa_1560, and Halsa_2086 possibly encode for putative Na^+^/H^+^ antiporter NhaC-like proteins. In addition, Halsa_0689 and Halsa_0691 possibly code for cation/proton antiporters. The gene that is present between these two, Halsa_0690, is a putative multiple resistance and pH regulation protein F gene. The two genes after Halsa_0691, (Halsa_0692 and Halsa_0693) possibly encode for subunits of a multicomponent Na^+^/H^+^ antiporter. Thus, many of these genes are likely involved with the maintenance of osmotic pressure and Halsa_0690 might be involved with pH regulation of the cell.

Besides potassium and sodium, other cations need to be transported into the cell. There are three copies, (Halsa_0666, Halsa_1667, and Halsa_2286) of a magnesium transporter for *Halanaerobium hydrogeniformans*. There is one putative gene for a cobalt transport protein (Halsa_1890). Halsa_1241 is a putative gene for a chromate transporter. Two putative zinc/iron permease genes are next to each other on the genome (Halsa_2161 and Halsa_2162). These cations, along with iron, would need to be taken up into the cell to serve as co-factors for enzymatic activity. Furthermore, one possible way that ammonium can enter the cell is through putative cation transporter Halsa_1351.

Another aspect that needs to be balanced between the cell and its haloalkaline environment is the anions, especially chloride. For example, *Halobacillus halophilus*, a low G+C, Gram-positive, moderately halophilic bacterium, has an absolute requirement for chloride (Saum et al., 2013). *Halanaerobium hydrogeniformans* possesses a putative Cl^-^ channel voltage-gated family protein (Halsa_0736) and an anion transporter (Halsa_0628) that can possibly transport chloride into the cell and help to achieve an anionic balance.

### SUMMARY

*Halanaerobium hydrogeniformans* is a unique bacterium that is ideally adapted to its haloalkaliphilic lake environment. It is capable of utilizing a variety of carbon sources and appears to possess the cell membrane transport systems to bring them into the cell. Once inside the cell, there is a complete Embden-Meyerhof pathway of glycolysis. However, the Kreb’s cycle is not complete. The organism relies on a number of fermentative metabolisms. It has been found to form acetate, formate, and hydrogen as fermentation products from simple sugars. It can also ferment glycerol, a widespread carbon source in saline environments. The bacterium also possesses transporters to bring in required metals and other ions. In addition to the metals required for enzymatic activity, the organism also possesses a variety of transporters that can bring in potassium and remove sodium to help to regulate the osmotic pressure. The Na^+^/H^+^ antiporters are important for both maintaining osmotic pressure and the pH of the cell. The organism also possesses a number of transposases. The transposases enable the organism to mobilize genes and affect gene regulation.

*Halanaerobium hydrogeniformans* has a number of similarities to *Halanaerobium praevalens* GSL^T^. Both organisms do not appear to possess formate-hydrogen lyase while they do appear to possess glycosyl transferases and fructose-specific phosphotransferase. On the other hand, the two organisms have a number of differences that are likely related to the environments, hypersaline vs. haloalkaline, where they were isolated from. *Halanaerobium praevalens* GSL^T^ possesses fewer genes for metabolism, such as the genes required for amino acid metabolism and cofactor and vitamin production. *Halanaerobium praevalens* GSL^T^ does not possess diguanylate cyclase with metal dependent phosphohydrolase genes or many of the metal-uptake proteins that *Halanaerobium hydrogeniformans* possesses. Furthermore, *Halanaerobium praevalens* GSL^T^ possesses less than a third of the number of transposase genes that *H. hydrogeniformans* does. The presence of these genes in *Halanaerobium hydrogeniformans* likely enables the organism to better tolerate the alkaline conditions, in addition to the saline conditions, and the metal content present in the sediments of Soap Lake. Furthermore, the transposases could provide genetic diversity that can lead to adaptive advantages for *Halanaerobium hydrogeniformans*.

## Conflict of Interest Statement

The author holds two patents on biohydrogen production by Halanaerobium hydrogeniformans. Elias, Mormile, Begemann, and Wall. A combined fossil fuel free process of lignocellulosic pretreatment with biological production, U.S. Patent No. US 8,148,133, Issued: April 3, 2012. Elias, Mormile, Begemann, and Wall. Fossil Fuel-Free Process of Lignocellulosic Pretreatment with Biological Hydrogen Production, U.S. Patent No. US 8,034,592 B2, Issued October 11, 2011.

## Footnotes

1 http://www.genome.jp/kegg-bin/show_pathway?has00010

2 http://www.genome.jp/kegg-bin/show_pathway?has00561
